# Assessing the biodiversity of rhizosphere and endophytic fungi in *Knoxia valerianoides* under continuous cropping conditions

**DOI:** 10.1186/s12866-024-03357-7

**Published:** 2024-06-07

**Authors:** Chunju Liu, Lei Zhang, Heng Li, Xiahong He, Jiahong Dong, Bin Qiu

**Affiliations:** 1 College of Plant Protection, Yunnan Agricultural University, Kunming, 650201 China; 2 Institute of Medicinal Plant Cultivation, School of Chinese Materia Medica, Academy of Southern Medicine, Yunnan University of Chinese Medicine, Kunming, 650500 China; 3 R&D center of Yunnan Yuntianhua Co., Ltd, Kunming, 650228 China; 4 Southwest Forestry University, Kunming, 650244 China

**Keywords:** *Knoxia valerianoides*, Continuous cropping, Rhizosphere fungi, Endophytic fungi, Physico-chemical properties

## Abstract

**Background:**

Rhizosphere and endophytic fungi play important roles in plant health and crop productivity. However, their community dynamics during the continuous cropping of *Knoxia valerianoides* have rarely been reported. *K. valerianoides* is a perennial herb of the family Rubiaceae and has been used in herbal medicines for ages. Here, we used high-throughput sequencing technology Illumina MiSeq to study the structural and functional dynamics of the rhizosphere and endophytic fungi of *K. valerianoides.*

**Results:**

The findings indicate that continuous planting has led to an increase in the richness and diversity of rhizosphere fungi, while concomitantly resulting in a decrease in the richness and diversity of root fungi. The diversity of endophytic fungal communities in roots was lower than that of the rhizosphere fungi. Ascomycota and Basidiomycota were the dominant phyla detected during the continuous cropping of *K. valerianoides*. In addition, we found that root rot directly affected the structure and diversity of fungal communities in the rhizosphere and the roots of *K. valerianoides*. Consequently, both the rhizosphere and endophyte fungal communities of root rot-infected plants showed higher richness than the healthy plants. The relative abundance of *Fusarium* in two and three years old root rot-infected plants was significantly higher than the control, indicating that continuous planting negatively affected the health of *K. valerianoides* plants. Decision Curve Analysis showed that soil pH, organic matter (OM), available K, total K, soil sucrase (S_SC), soil catalase (S_CAT), and soil cellulase (S_CL) were significantly related (*p* < 0.05) to the fungal community dynamics.

**Conclusions:**

The diversity of fungal species in the rhizosphere and root of *K. valerianoides* was reported for the first time. The fungal diversity of rhizosphere soil was higher than that of root endophytic fungi. The fungal diversity of root rot plants was higher than that of healthy plants. Soil pH, OM, available K, total K, S_CAT, S_SC, and S_CL were significantly related to the fungal diversity. The occurrence of root rot had an effect on the community structure and diversity of rhizosphere and root endophytic fungi.

## Introduction

The rhizosphere is defined as a narrow area of soil around the roots that has the most significant effect on plant nutrition and growth [1, 2]. Fungal communities colonize both the plants and the rhizosphere soil affecting plant’s fitness in various ways, such as influencing nutrient uptake, inducing soil-borne diseases, and affecting the activity of plant pathogens [3]. Interactions between plant roots and rhizosphere microbiomes collectively form the plant-root microorganism complex [4]. Some agronomic practices can affect plant-microbial interactions, including continuous cropping [5–7], crop rotation [8], intercropping [9], irrigation and fertilization [10, 11], as well as the application of pesticide-fertilizer combinations [12]. Continuous cropping is widely practiced in Chinese agriculture, which involves the cultivation of the same crop in the same soil year after year [13]. Continuous cropping for many years causes loss in crop yields, deterioration in quality, increased susceptibility to pests and diseases, and reduction in soil fertility [3]. The harmful effects of continuous cropping have been reported on various rhizome medicinal plants, including *Salvia miltiorrhiza* [14], *Panax notoginseng* [15], and *Coptis chinensis* [16].

*Knoxia valerianoides*, also known as ‘Zi Daji’ in China, is a perennial herb belonging to the family Rubiaceae, and its root is a traditional Chinese medicine (TCM) owing to its medicinal properties [17]. The roots of *K. valerianoides* are the main ingredient in the Chinese patent medicine Zijinding [18]. The major organic compounds in the extracts of *K. valerianoides* are anthraquinones and triterpenoids [19, 20]. Studies have demonstrated that continuous cropping leads to the decrease in the yield and quality of *S. miltiorrhiza* [14], which may be attributed to the composition change of the rhizosphere soil microbial community [21]. Changes in soil microflora are considered the primary cause of yield decline in rhizome medicinal plants under continuous cropping conditions [22]. Root rot is a common disease affecting the cultivation of various root and rhizome medicinal plants, and has been reported on numerous occasions [23, 24]. In recent years, with the expansion of Chinese herbal medicine cultivation, root rot has been reported to increase with each passing year in continuous planting conditions. *P. notoginseng* is particularly susceptible to root rot disease, with an annual incidence ranging from 5 to 20%, causing severe losses sometimes up to 70% [25]. Similarly, in Canada 20-30% of ginseng crops are lost annually to root rot [26]. Studies have shown that most pathogens of root rot are fungi, including *Fusarium oxysporum*, *F. solani*, and *Rhizoctonia solani* [27–29], with *Fusarium* as the predominant pathogenic factor.

Rhizosphere fungi are involved in important ecological processes and thus are key players in the promotion of plant growth and maintenance of plant health [30, 31]. Some rhizosphere fungi are associated with pathogen growth inhibition and thus act as biocontrol agents; however, imbalances in rhizosphere fungal community structure have been associated with the development of root rot [32, 33]. Furthermore, endophytic fungi, characterized by their non-pathogenic nature, have shown promise as biological control agents [34]. The presence and abundance of endophytic fungi significantly influence the overall health of host plants, primarily through their positive impact on the plant’s immune system [35, 36]. Recent studies have indicated that continuous cropping practices can disrupt the balance of plant root microbial complex by the accumulation of pathogenic fungi in the rhizosphere [3, 37].

There are several reports exploring the plant root microbial complex of rhizome medicinal plants; however, there is no such report on *K. valerianoides*. In this study, we aimed to fill this knowledge gap. The primary objective was to investigate the diversity and composition of fungal communities within the plant root microbial complex of *K. valerianoides* using high-throughput sequencing technology. Second objective was to characterize rhizosphere and endophytic fungal communities of healthy and infected *K. valerianoides* under continuous cropping. Additionally, we aimed to analyze the soil physico-chemical properties and enzymatic activity, and determine their relationship with rhizosphere and endophytic fungal community structures. The overarching goal of this study was to gain insights into the pathogenesis of *K. valerianoides* root rot.

## Materials & methods

### Study site and experimental design

The field experiment was performed in Xiangyun County, Dali Bai Autonomous Prefecture of Yunnan Province (25°25′N, 100°40′E), a prominent region for the cultivation of *K. valerianoides* in China. In August 2022, root rot was observed on *K. valerianoides* root with about 15% incidence. The rhizosphere soil of 1, 2, and 3-years old *K. valerianoides* plants were collected by using a diagonal method [5]. A total of 6 plots were selected for sampling. Within each sample plot, rhizosphere soil samples of 5 healthy or root rot-infected *K. valerianoides* plants were combined to make a single sample, and the unplanted soil of the same plot served as control. The average depth of the main root and fibrous root of *K. valerianoides* was determined to be 0–30 cm. Consequently, sampling was conducted at a depth of 0–30 cm. During sampling, the plants were carefully excavated, and the loose soil that naturally fell off was discarded. The soil left on the root surface was considered the rhizosphere soil [5]. After collection, each sample was sieved using a 4 mm soil sieve, and the remaining root sample was quickly placed in a dry ice box storage. The sampling sites were all abandoned recreational land before the planting of *K. valerianoides*; after planting, they were managed using a mixed model of organic fertiliser and chemical fertiliser (continuous planting period of 3–4 years).

The root surface disinfection steps are as follows: The root surface was rinsed with flowing tap water, followed by three rinses with sterile distilled water. The root of *K. valerianoides* was then divided into small sections with a sterile scalpel for surface disinfection. Each tissue sample was immersed in 70% ethanol for 4 min, washed with fresh 2% NaClO solution for 1–2 min, subsequently immersed in 70% ethanol for 1 min, and finally rinsed three times with sterile distilled water [38].

Five replicated soil or root samples were combined to form a single sample. These samples included healthy rhizosphere soil from 1-year-old (S_H1), 2-year-old (S_H2), and 3-year-old (S_H3) *K. valerianoides* plants. Similarly, root rot affected rhizosphere soil from 2-year-old (S_D2), and 3-year-old (S_D3) *K. valerianoides* plants. A sample from unplanted soil of the same plot (S_CK) was used as a control. Additionally, endophyte samples of healthy roots from 1-year-old (R_H1), 2-year-old (R_H2), and 3-year-old (R_H3) *K. valerianoides* plants were collected, along with endophytic samples of root rot affected 2-year-old (R_D2), and 3-year-old (R_D3) *K. valerianoides* plants; while samples from 1-year-old plants (R_H1) were used as control. All the samples were stored at -80℃ until further analyses.

### Soil physico-chemical properties and enzyme activity

The physico-chemical properties and enzyme activity of all soil samples were analyzed following established protocols. Soil pH was measured using a glass electrode pH meter (PHS-3E, China) in a 2.5:1 water: soil (v/w) suspension. Soil organic matter (OM) content was determined by K_2_Cr_2_O_7_ redox titration [39], soil hydrolytic nitrogen (HN) was quantified by alkaline hydrolysis, while soil available phosphorus (AP) was extracted with sodium bicarbonate and determined by molybdenum blue method [40]. The measurement of soil available potassium (AK) followed standard procedures outlined by MC Clean [41]. Established protocols were followed to measure total nitrogen (TN) [42], total phosphorus (TP), and total potassium (TK) [43] in the soil samples. To determine the enzymatic activity of soil samples; namely soil catalase (S_CAT), soil urease (S_UE), soil sucrase (S_SC), and soil cellulase (S_CL), specific kits (G0303F, G0301F, G0302F, G0308F, Grace) were employed. S_CAT, S_UE, and S_SC are soil extracellular enzymes whose activities are important bioactivity indicators for soil quality tests. S_CL play an important role in the degradation and decomposition of organic matter in soil. The assay procedures were performed following the kit instructions, and the measurements were taken using an ultraviolet spectrophotometer (Shanghai Jinghua 754).

### DNA extraction, PCR amplification, and sequencing

Total microbial genomic DNA was extracted from the rhizosphere soil, blank control soil samples, and plant root samples using the PowerSoil Pro DNA Kit (Qiagen, Hilden, Germany) following the manufacturer’s instructions. The quality and concentration of DNA were assessed by 1.0% agarose gel electrophoresis and a NanoDrop® ND-2000 spectrophotometer (Thermo Scientific Inc., USA) and kept at -80 ℃ until further analyses. For the amplification of the hypervariable region ITS of the fungal ITS gene, primer pairsITS1-F (5’- CTTGGTCATTTAGAGGAAGTAA-3’) and ITS2-R (5’-GCTGCGTTCTTCATCGATGC-3’) were utilized with an ABI GeneAmp® 9700 PCR thermocycler (ABI, CA, USA) [44]. The PCR reaction mixture consisted of 5×Fast Pfu buffer (4 µL), 2.5 mM dNTPs (2 µL), 5 µM each primer (0.8 µL), Fast Pfu polymerase (0.4 µL), template DNA (10 ng), and ddH_2_O was added to make a final volume of 20 µL. The PCR amplification procedure involved initial denaturation at 95 ℃ for 3 min, followed by 27 cycles of denaturing at 95 ℃ for 30 s, annealing at 55 ℃ for 30 s, and extension at 72 ℃ for 45 s, with a final single extension at 72 ℃ for 10 min, and subsequent cooling at 4 ℃. All samples were amplified in triplicate, and the resulting amplicons were extracted from 2% agarose gel and purified using the AxyPrep DNA Gel Extraction Kit (Axygen Biosciences, Union City, CA, USA), following the manufacturer’s instructions. The quantification of the extracted amplicon was performed using a Quantus™ fluorescence meter (Promega, USA). The sequencing library was prepared by Illumina according to their established protocols. Subsequently, the prepared library underwent qualification to assess its quality and integrity. For sequencing, the Illumina MiSeq PE300 platform (Illumina, San Diego, USA) was employed, and the sequencing service was entrusted to Shanghai Majorbio Bio-Pharm Technology Co., Ltd., following their standard procedures.

### Data processing and statistical analyses

The sequencing raw reads obtained from the Illumina MisSeq PE300 platform were subjected to quality control using fastp (version 0.19.6) to remove low-quality reads. The remaining high-quality reads were further processed for merging using FLASH (version 1.2.11) to obtain longer sequences [45, 46]. The optimized sequences were then clustered into operational taxonomic units (OTUs) using UPARSE 7.1 with a 97% sequence similarity threshold [47]. To account for variations in sequencing depth, the number of sequences in each sample was normalized to 20,000 reads, ensuring comparable coverage among samples while maintaining an average sequence coverage of 99.9%. The taxonomic classification of each representative OTU sequence was performed using the RDP Classifier (version 2.11, http://sourceforge.net/projects/rdp-classifier/) against the ITS fungi database (release 8.0, http://unite.ut.ee/index.php) using a confidence threshold of 0.7. Alpha diversity metrics including Sobs, Chao, Shannon, Simpson, and Coverage were calculated by mothur software (version 1.30.2, http://www.mothur.org/wiki/Calculators), and the difference in Alpha diversity between groups was analyzed by Student’s t-test [48]. Statistical analyses and data visualization were performed in R language (version 3.3.1).

Non-metric multidimensional scaling (NMDS) analysis based on the abund-jaccard distance algorithm was employed to explore similarities in microbial community structure among samples, and a heatmap diagram was generated to visualize the differences in species composition. Additionally, Decision Curve Analysis (DCA) was performed to investigate the impact of soil environmental factors on the fungal community structure in the rhizosphere roots. Data correlation analysis was carried out using SPSS 19.0.

## Results

### Soil physico-chemical properties and enzyme activity

The analysis of soil physico-chemical properties and enzyme activity revealed notable effects of continuous cropping on the soil. Specifically, the pH of the continuous cropping soil showed a significant increase (*p* < 0.05) compared to the unplanted soil (S_CK). Furthermore, the continuous cropping soil exhibited significantly higher levels of OM, hydrolyzable nitrogen (N), available phosphorus (P), total nitrogen (N), and total phosphorus (P) in comparison to the unplanted soil. Additionally, the enzyme activities of soil sucrase (S_SC) and soil urease (S_UE) were significantly higher in the continuous cropping soil than in the unplanted soil. S_SC increased with the increase of planting years. Available potassium (K) and S_UE showed that healthy plants were higher than infected plants, while S_CAT, pH and S_CL showed the opposite (Table 1).

**Table 1:** Soil physico-chemical properties and enzyme activity of rhizospheric soils in Xiangyun county, China (*n* = 6). Different letters in the same column indicate significant differences at *p* < 0.05

Sample	pH	Organic matter (g/kg)	Hydrolytic N (mg/kg)	Available P (mg/kg)	Available K (mg/kg)	Total N (g/kg)	Total P (g/kg)	Total K (g/kg)	S_CAT (µmol/h/g)	S_SC (mg/d/g)	S_UE (µg/d/g)	S_CL (µg/d/g)
S_D2	7.75 ± 0.15a	20.08 ± 1.22b	105.00 ± 10.00b	24.93 ± 4.29a	168.17 ± 49.67bc	2.04 ± 0.08b	1.07 ± 0.03ab	13.07 ± 0.52a	154.02 ± 12.20a	6.80 ± 1.04a	96.48 ± 9.45a	162.95 ± 49.66a
S_D3	7.04 ± 0.35c	25.62 ± 1.69a	136.00 ± 14.04a	24.88 ± 4.54a	312.67 ± 90.23a	2.10 ± 0.17b	1.10 ± 0.06ab	10.58 ± 0.41b	94.72 ± 19.52b	7.85 ± 1.20a	99.10 ± 5.19a	131.87 ± 43.55ab
S_H1	6.76 ± 0.22d	24.42 ± 2.56a	121.17 ± 17.65ab	23.42 ± 3.57a	428.50 ± 80.01a	2.11 ± 0.23b	1.03 ± 0.06b	9.87 ± 0.55c	82.18 ± 9.67bb	5.50 ± 0.84b	102.25 ± 6.77a	199.70 ± 48.32a
S_H2	7.41 ± 0.14b	19.47 ± 2.52b	104.50 ± 15.58b	24.18 ± 4.47a	185.00 ± 13.56b	2.38 ± 0.16a	1.12 ± 0.07a	13.08 ± 0.50a	146.13 ± 38.89a	6.93 ± 0.85a	98.17 ± 7.24a	72.85 ± 15.46c
S_H3	6.81 ± 0.21 cd	26.57 ± 3.23a	125.83 ± 21.54a	25.03 ± 4.44a	377.00 ± 71.03a	2.25 ± 0.17ab	1.11 ± 0.08ab	10.32 ± 0.38bc	64.15 ± 13.37c	7.83 ± 0.77a	101.03 ± 11.07a	93.63 ± 24.63bc
S_CK	6.33 ± 0.23e	14.20 ± 2.69c	90.17 ± 14.77c	9.65 ± 2.80b	117.17 ± 27.90c	1.65 ± 0.22c	0.90 ± 0.10c	10.93 ± 0.75b	64.77 ± 24.51c	4.34 ± 0.98c	85.02 ± 7.87b	86.33 ± 13.10c

### Composition and α-Diversity of rhizosphere and endophytic fungi

**Fig. 1:**
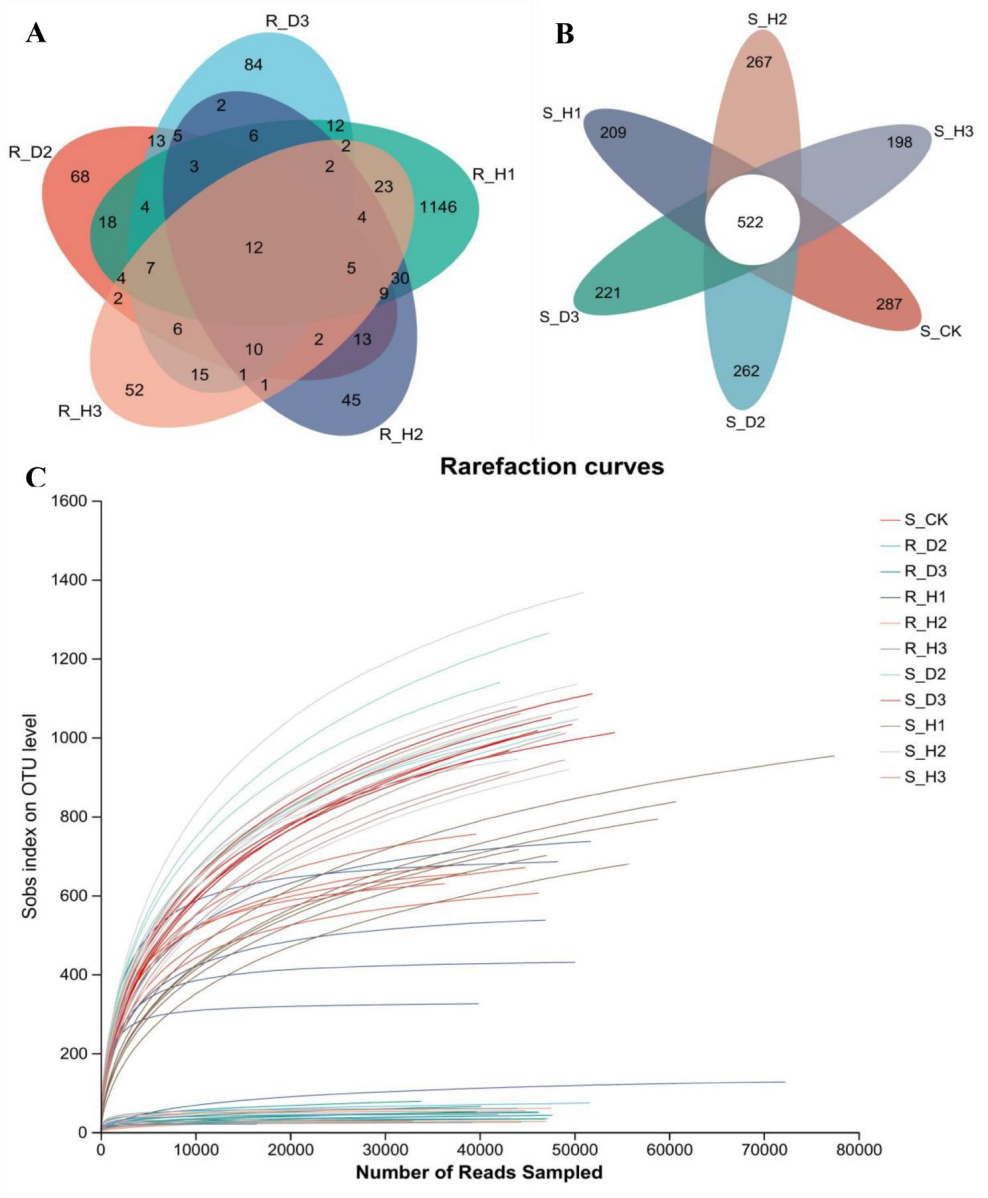
A and B Venn diagrams at the OTU level in root (**A**) and soil (**B**) groups. Rarefaction curves(**C**) of fungal communities based on observed operational taxonomic units (OTUs) for 11 plant and soil samples under a *Knoxia valerianoides* continuous cropping system. Repeat 6 times for each sample

Composition and α-diversity analyses were performed to investigate the fungal communities in the rhizosphere and endophytic compartments. High-throughput sequencing was performed on collected samples, including six rhizosphere soil fungi (S_H1, S_H2, S_H3, S_D2, S_D3, S_CK) and five root endophytic fungi samples (R_H1, R_H2, R_H3, R_D2, R_D3) with six biological replicates per sample. A total of 3,211,327 ITS sequences were obtained after quality control, resulting in 769,901,943 bases with an average length of 239 bp. Subsequent normalization analysis yielded 33,872 sequences per sample, generating 5,312 OTUs. Of these, 1,953 OTUs were found in the root samples of *K. valerianoides*, 522 OTUs were shared among the rhizosphere soil samples of *K. valerianoides*, and 12 OTUs were shared among the root samples of *K. valerianoides* (Fig. 1A and B).

The dilution curve was plotted using randomly selected sequencing data to assess Alpha diversity, with sequencing data as the abscissa and Alpha diversity index value as the ordinate (Fig. 1C). The curve of each sample displayed a plateau, indicating that the obtained reads adequately represent the fungal community’s diversity, and the coverage of all samples exceeded 99.31%.

Alpha diversity was estimated by calculating richness indices such as Sobs, Chao, Ace, Simpson, and others (Table 2). Overall, rhizosphere soil fungi exhibited higher richness and diversity indices compared to root endophytic fungi. Within the same planting period, the diversity indexes (Sobs, Chao, Simpson, and Ace) of endophytic fungi in root rot-infected roots were higher compared to the healthy plants, with R_D3 showing higher diversity than R_D2. Healthy plant R_H1 showed higher richness and diversity, while the Ace index decreased gradually with the increasing continuous cropping period. Similar patterns were observed for rhizosphere fungal diversity indices in relation to the root rot disease. The diversity indexes Ace and Chao of the healthy rhizosphere fungi increased with the age of the plant and were significantly higher *(p* < 0.05) than S_H1. The rhizosphere fungal diversity indices of root rot-affected plants, including Sobs, Ace, and Chao were significantly higher than those of S_CK. The field survey found that the incidence of 1–3 year old *K. valerianoides* root rot gradually increased with the increase of planting years, which was basically consistent with the change of fungi diversity with years.

**Table 2:** The diversity indexes of endophytic fungi and rhizosphere fungi were tested by LSD in One-way ANOVA at Genus level (*n* = 6). Different letters in the same column indicate significant differences at *p* < 0.05 among the samples from same source

Source of sample	Sample	Sobs	Shannon	Simpson	Ace	Chao	Coverage
Root	R_D2	29.83 ± 8.04b	1.14 ± 0.32b	0.45 ± 0.16ab	32.18 ± 8.97b	31.05 ± 8.50b	0.9999
	R_D3	32.50 ± 6.89b	0.98 ± 0.50b	0.57 ± 0.24a	47.67 ± 23.09b	39.64 ± 10.97b	0.9998
	R_H1	183.17 ± 74.41a	3.06 ± 1.44a	0.21 ± 0.37b	188.01 ± 73.34a	189.53 ± 71.46a	0.9997
	R_H2	24.67 ± 8.76b	0.72 ± 0.71b	0.70 ± 0.31a	24.84 ± 8.80b	24.67 ± 8.76b	1.0000
	R_H3	27.17 ± 9.11b	1.18 ± 0.42b	0.49 ± 0.15ab	24.31 ± 14.59b	27.33 ± 9.46b	1.0000
Soil	S_D2	330.00 ± 19.43a	4.01 ± 0.14a	0.04 ± 0.01b	369.98 ± 28.58a	369.71 ± 28.26a	0.9985
	S_D3	325.00 ± 6.48a	3.75 ± 0.12ab	0.05 ± 0.01b	366.07 ± 16.00a	362.08 ± 10.76a	0.9984
	S_H1	237.17 ± 14.63b	2.60 ± 0.25c	0.17 ± 0.05a	284.32 ± 20.86b	285.06 ± 24.56b	0.9984
	S_H2	316.17 ± 33.76a	3.75 ± 0.41ab	0.07 ± 0.06b	351.46 ± 40.35a	348.25 ± 40.58a	0.9986
	S_H3	314.33 ± 17.42a	3.69 ± 0.20b	0.05 ± 0.01b	355.08 ± 24.35a	356.85 ± 34.61a	0.9984
	S_CK	251.33 ± 12.08b	3.74 ± 0.20ab	0.04 ± 0.01b	261.14 ± 13.03b	266.99 ± 16.70b	0.9994

### Structure of the fungal communities

**Fig. 2:**
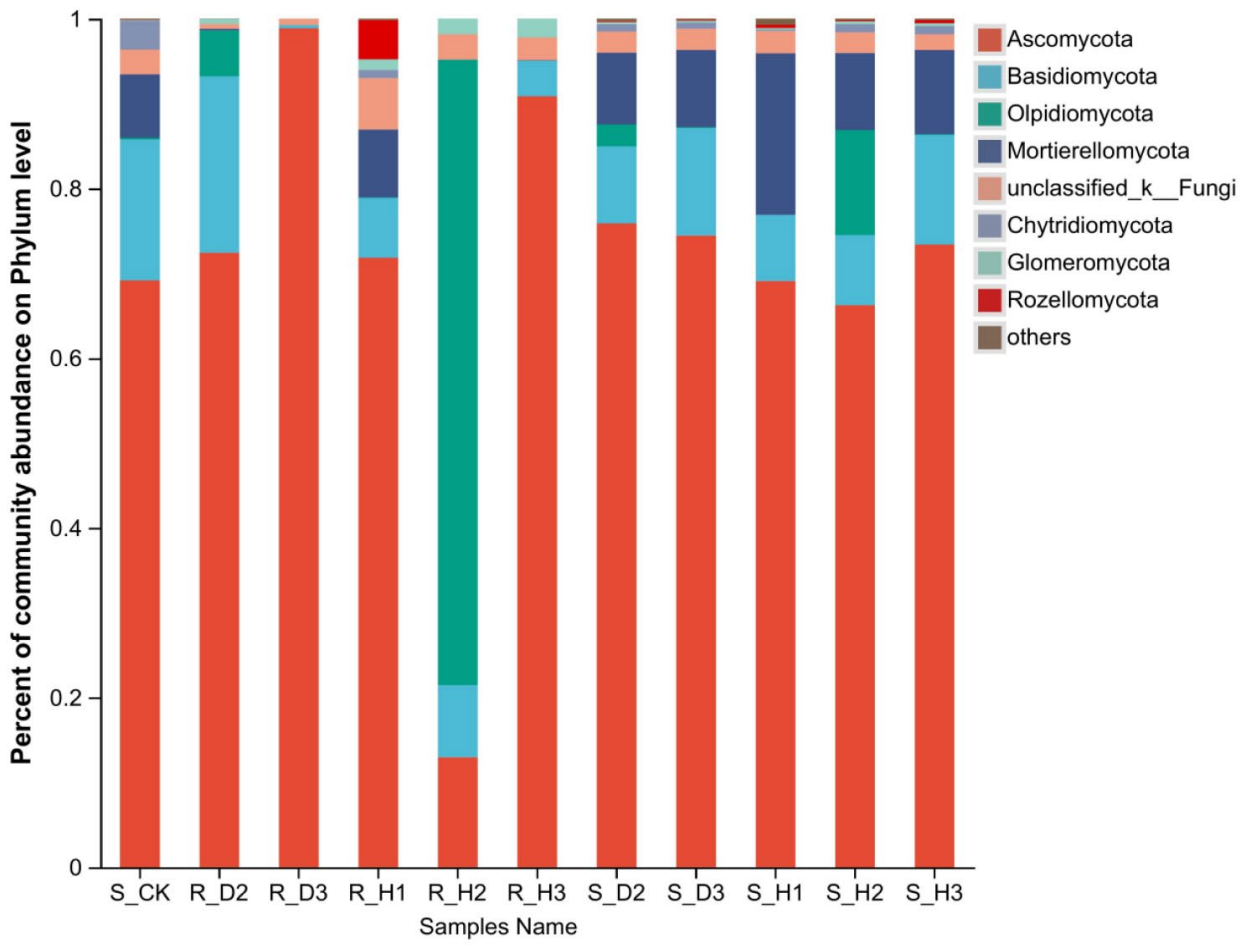
Relative species abundance at the phylum level in fungi communities, visualized horizontally. Based on the species annotation results, we choose to examine the distribution of relative species abundance among the top-ranked phyla; the relative abundance distribution of the top ten species is shown

The structure of the fungal communities was investigated, revealing a total of 883 genera, 365 families, 152 orders, 61 classes, and 16 phyla in the rhizosphere soil and root samples of *K. valerianoides*. As illustrated in Fig. 2, the dominant phyla were Ascomycota, Basidiomycota, Olpidiomycota, and Mortierellomycota. Notably, the abundance of Ascomycota in R_H3 and R_D3 was the highest, accounting for 90.86% and 98.88%, respectively.

**Fig. 3:**
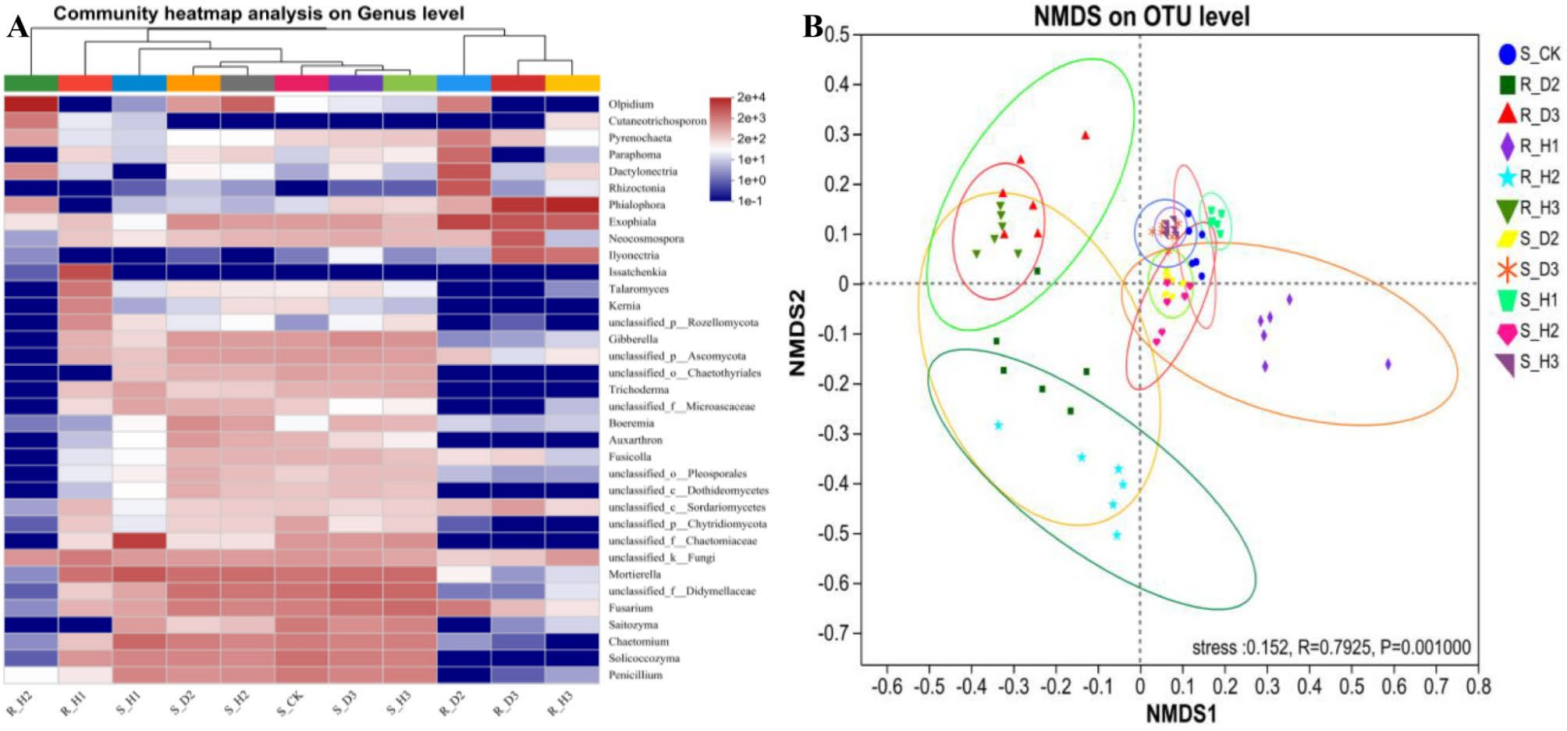
The top 35 most abundant fungal communities across 11 plant and soil samples (**A**) and non-metric multidimensional scaling (NMDS) based on the operational taxonomic unit (OTUs) composition (**B**). Repeat 6 times for each sample

Hierarchical cluster analysis was performed on the top 35 genera based on the fungal abundance in 11 plant and soil samples, and the results are presented in Fig. 3A. The analysis revealed that all rhizosphere soil samples were clustered together into one category, while the root samples were divided into three distinct categories. The relative abundance of certain fungal genera in soil and root samples differed significantly across different continuous cropping years. *Solicoccozyma* (8%) was the dominant genus in S_CK, but its relative abundance decreased with increasing continuous cropping time. *Mortierella* and *Didymellaceae* were dominant genera in S_H3 (10%), S_D2 (8%), and S_D3 (9%, 12%), with S_D3 exhibiting significantly higher abundance than S_D2. *Chaetomiaceae* was the dominant genus in S_H1 (33%), *Olpidium* in S_H2 (12%) and R_H2 (73%), *Phialophora* in R_H3 (64%) and R_D3 (41%), *Exophiala* in R_D2 (28%), and *Issatchenkia* in R_H1(21%).

To visually represent the differences between different treatments, NMDS analysis based on abund-jaccard dissimilarity distance was performed on the sample data. The results demonstrated significant differences among different samples (*R* = 0.7925, *p* = 0.001), and the stress value was 0.152, indicating that continuous cropping exerted a significant impact on the composition of the fungal community (Fig. 3B).

### Correlation between soil properties, enzyme activity, and the fungal communities

**Fig. 4:**
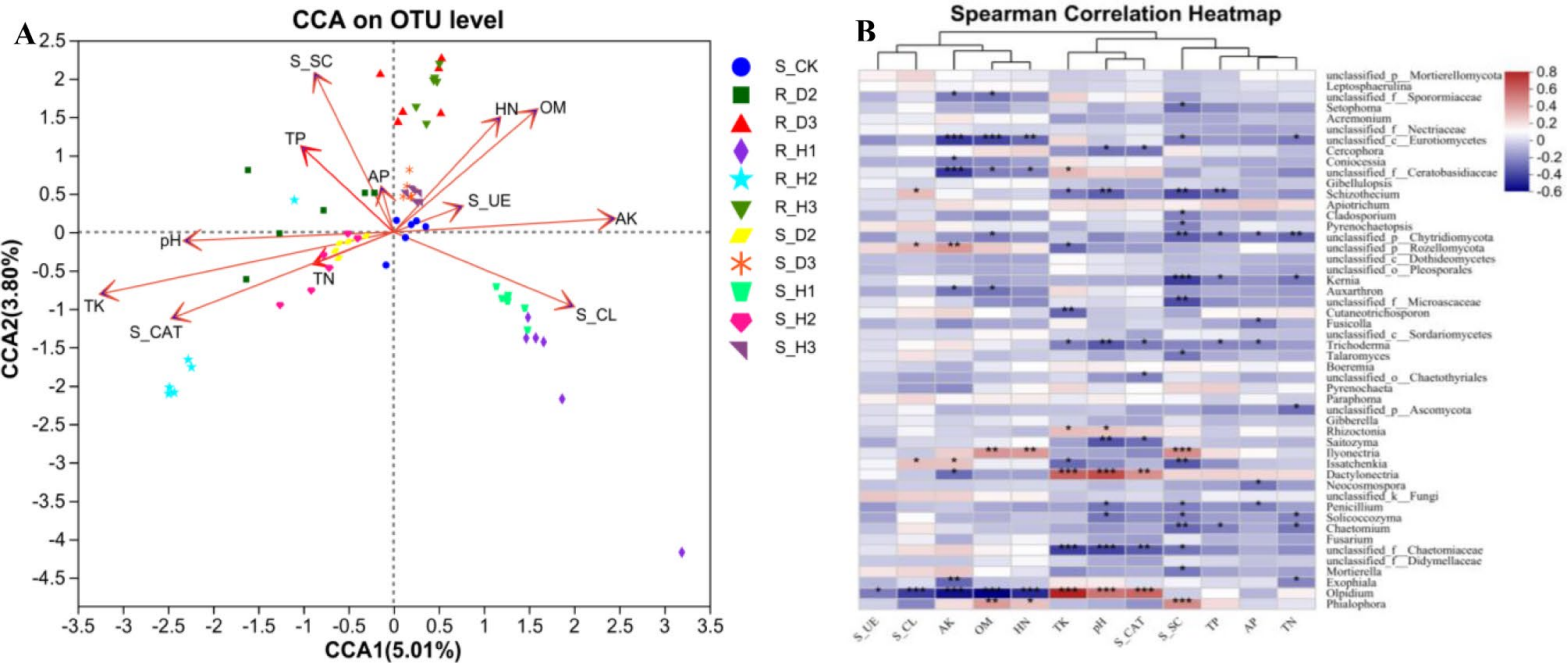
Canonical correspondence analysis (CCA) between the soil properties, enzyme activity and fungal community (**A**). Spearman correlation heatmap between the soil properties, enzyme activity and fungal community (**B**)

The correlation between soil properties, enzyme activity, and fungal community composition was examined using DCA. The results revealed that the response of fungal community composition to soil properties followed a unimodal model (Axis_lengths = 8.05). Consequently, Canonical Correspondence Analysis (CCA) was employed to investigate the relationship between the fungal community and soil environmental factors. It was observed that the soil environmental factors such as pH, total P, total K, total N, and S_CL exhibited correlations among themselves, as did OM, hydrolytic N, available K, S_UE, and S_CL. Furthermore, pH (*p* = 0.001), OM (*p* = 0.001), available K (*p* = 0.001), total K (*p* = 0.001), total P (*p* = 0.018), S_CAT (*p* = 0.001), S_SC (*p* = 0.001) and S_CL (*p* = 0.001) demonstrated significant correlations with fungal community structure (Fig. 4A). The Spearman correlation heatmap indicated that the relative abundance of the genus *Olpidium* exhibited significant negative correlations with S_UE, S_CL, available K, OM and hydrolytic N while displaying positive correlations with total K, pH, and S_CAT. Moreover, the relative abundance of the genus *Dactylonectria* showed a significant positive correlation with total K, pH, and S_CAT. Genus *Phialophora* exhibited significant positive correlations with OM, hydrolytic N, and S_SC (Fig. 4B).

## Discussion

The sustainable production of *K. valerianoides* necessitates a profound understanding of the fungal community’s structure and diversity. In this study, we employed high-throughput sequencing technology to assess the impact of continuous planting on the rhizosphere soil and root endophytic fungal communities of *K. valerianoides*. Our findings revealed that continuous cropping exerted a substantial influence on the diversity, structure, and composition of rhizosphere soil and endophytic fungal communities. Following continuous cropping, the Alpha diversity indices of Sobs, Ace, Chao, and Simpson exhibited an increase in the rhizosphere soil, implying that continuous cropping of *K. valerianoides* enhanced the diversity and richness of fungi in rhizosphere soil. This observation is consistent with previous studies involving *P. notoginseng* where, the diversity of fungi in the rhizosphere soil increased with the increase of continuous cropping period [37]. Conversely, the diversity index Ace and Chao of endophytic fungi in healthy plant roots exhibited a gradual decrease with increasing period of continuous cropping, along with a decline in the Shannon index. Root rot has traditionally been considered the primary concern in continuous cropping practices for cultivating rhizome medicinal plants [49]. In comparison to the infected plants, we observed relatively lower fungal diversity in healthy plants and rhizosphere soil samples. Consequently, the reduction in the diversity of rhizosphere and endophytic fungi, especially the decrease in beneficial taxa [50], may contribute to the occurrence of diseases during the process of continuous cropping.

Continuous cropping of *K. valerianoides* significantly influenced the community composition of rhizosphere soil fungi. Ascomycota and Basidiomycota, known as key decomposers among soil fungal communities [49], were identified as the dominant phyla in all rhizosphere soil samples, confirming their ecological importance. This observation is consistent with the previous findings [30]. At the genus level, *Fusarium* and *Gibberella* exhibited an increase in abundance with prolonged continuous cropping periods. *Fusarium* species are notorious plant pathogens [51], including important pathogenic species of *F. oxysporum* and *F. solani* [27]. Prior studies have indicated that the disturbance of rhizosphere microbial communities, particularly the proliferation of potential pathogens, could contribute to the transition of the rhizosphere microenvironment from ‘health’ to ‘disease’ [52]. Thus, the observed changes in rhizosphere soil fungal communities, including the increased abundance of *Fusarium* and *Gibberella*, suggest a potential association between continuous cropping and the development of pathogenic conditions.

In recent years, the significance of endophytic fungi in plant tissues for plant health has garnered considerable attention. During continuous cropping, the diversity of endophytic fungi in roots was found to be lower than that in rhizosphere soil. Furthermore, a decline in endophytic fungal diversity indices Chao, Ace, and Sobs was observed in infected root samples as the duration of planting increased, indicating a reduction in endophytic fungal abundance. Ascomycota and Basidiomycota were identified as the dominant phyla in both healthy and infected *K. valerianoides* plants, mirroring the fungal distribution observed in rhizosphere soil. Ascomycetes was the most prevalent taxon, as it plays an important ecological role as a decomposer [53]. Previous studies have demonstrated that endophytic fungi primarily originate from soil fungi and gain entry into plants through roots, stems, and leaves [37].

Given that *K. valerianoides* roots are used in traditional medicine, it is crucial to note that certain coexisting endophytic fungi can significantly impact the formation of plant metabolites, consequently influencing the quality of medicinal plant raw materials [54]. Similarly, in case of marijuana (*Cannabis sativa* L.), the plants with pathogen-infected roots produced significantly reduced crop yield and quality [55]. At the genus level, there was a notable increase in the abundance of *Fusarium*, *Pyrenochaeta*, and *Exophiala* in root rot-infected samples. The proliferation of pathogens associated with root rot, such as *F. oxysporum*, *F. solani*, and *Phytophthora cactorum*, might have contributed to the reduction in endophytic fungi. This may be related to the fact that *K. valerianoides* planting improves soil organic matter content. These findings further substantiate the long-standing recognition of the genus *Fusarium* as an important plant pathogen [51]. Collectively, these outcomes serve as a foundation for future investigations concerning microbial community changes in *K. valerianoides* and their potential role as an indicator of plant health during continuous cropping.

The comprehensive changes in soil properties are intimately associated with continuous cropping challenges and crop productivity [22, 56]. Prolonged monoculture practices have had a detrimental impact on sustainable productivity, leading to a decrease in soil pH and OM content [57]. Long-term fertilization practices (32 years) can influence soil enzyme activity as well as physical and chemical properties [58]. Previous studies have indicated a correlation between the occurrence of ginseng root rot OM, available potassium, alkali-hydrolyzed nitrogen, pH, sucrase, and catalase activity in rhizosphere soil [59].

Microbial diversity encompasses the species composition and interspecific variations within microbial communities, reflecting the richness of soil microbial life, and serving as an indicator of soil quality and restoration potential [60]. Soil enzymes are an important biological indicator of the prevalence of material and energy metabolism in soil and soil quality. S_CAT, S_UE, S_SC and S_CL play important roles in the cycling and transformation of soil nutrients such as nitrogen and carbon, as well as in plant protection [61]. The S_UE activity of infected plants was lower than that of healthy plants, which was consistent with the results of previous studies [62]. In our study, soil pH, OM, available K, total K, S_CAT, S_SC, and S_CL exhibited the strongest correlations with fungal community structure. Soil pH has a profound influence on the chemical and physical properties of soil and serves as a primary factor shaping the microbial community composition in rhizosphere soil [63]. Notably, the rhizosphere soil of root rot-infected *K. valerianoides* exhibited higher pH levels compared to healthy soil. Furthermore, HN played a crucial role in fungal community composition under continuous cropping. The nitrogen fertilization system significantly impacts the composition of the soil fungal community, and the application of organic fertilizer can mitigate the abundance of soil fungal pathogens such as *Fusarium* [64]. Moreover, low irrigation practices can enhance soil enzyme activity [65], while high saline-alkali stress leads to a decrease in soil enzyme activity [66]. In our study, a significant correlation was observed between soil pH and S_CL, which may also contribute to the occurrence of root rot of *K. valerianoides*. It is speculated that pH and S_CL could be important predictors of fungal community composition in continuous cropping soil.

## Conclusions

In conclusion, continuous cropping of *K. valerianoides* significantly altered the structure and diversity of both the rhizosphere soil and root endophytic fungal communities. Continuous cropping led to an increase in the abundance of pathogenic fungi, consequently, promoting soil-borne diseases in *K. valerianoides*. The rhizosphere soil fungal community of root rot-infected plants exhibited higher richness compared to the control (S_CK). Severe root rot infection displayed a negative effect on the diversity of rhizosphere and root fungal communities. Moreover, continuous cropping resulted in a significant increase in soil pH, OM, hydrolyzable nitrogen, available phosphorus, total N, total P, and soil urease activity. Additionally, soil sucrose activity showed an increasing trend with the duration of the continuous cropping period. It is a compelling evidence to suggest important relationships of soil physico-chemical properties and enzyme activity with the structural dynamics of fungal communities. Further investigations on the rhizosphere and endophytic fungi of both healthy and infected plants are needed for comprehensive understanding of pathogenesis of root rot disease in *K. valerianoides*.

## Data Availability

Original data have been deposited into the NCBI SRA database with the accession number PRJNA981433.
